# Isolation and Characterization of *Gardnerella* Phage vB_Gva_AB1, a Bacteriophage Infecting a Clinical Strain of Gardnerella vaginalis

**DOI:** 10.1128/MRA.00053-21

**Published:** 2021-03-25

**Authors:** Alexia Bordigoni, Sonia Bouchard, Christelle Desnues

**Affiliations:** a Aix-Marseille Université, IRD 198, Assistance-Publique des Hôpitaux de Marseille, UMR Microbes, Evolution, Phylogeny and Infections (MEPHI), IHU Méditerranée Infection, Marseille, France; b Aix-Marseille Université, Université de Toulon, CNRS, IRD, Mediterranean Institute of Oceanography, UM 110, Marseille, France; Loyola University Chicago

## Abstract

Gardnerella vaginalis is the presumed causative agent of bacterial vaginosis. Here, we describe the complete genome sequence of *Gardnerella* phage vB_Gva_AB1, induced from a vaginal bacterial strain from a woman suffering with bacterial vaginosis. The phage double-stranded DNA (dsDNA) genome is 50,268 bp long with a GC content of 39.55% and contains 62 predicted open reading frames.

## Abstract (precis)

Gardnerella vaginalis is the presumed causative agent of bacterial vaginosis. Here, we describe the complete genome sequence of *Gardnerella* phage vB_Gva_AB1, induced from a vaginal bacterial strain from a woman suffering with bacterial vaginosis. The phage double-stranded DNA (dsDNA) genome is 50,268 bp long with a GC content of 39.55% and contains 62 predicted open reading frames (ORFs).

## ANNOUNCEMENT

Gardnerella vaginalis is an anaerobic bacterium considered one of the etiological agents of bacterial vaginosis (BV) (1, 2). Conventional treatment consists of clindamycin or metronidazole intake (3) with frequent relapses (4, 5). Probiotics are used to replace or complement traditional therapy (6), but their effects on BV are controversial (7–9). Phagotherapy could be another alternative treatment to restore vaginal flora (10). The genome of Gardnerella vaginalis strains contains a multitude of prophage sequences (11), but so far, none have been isolated.

**FIG 1:**
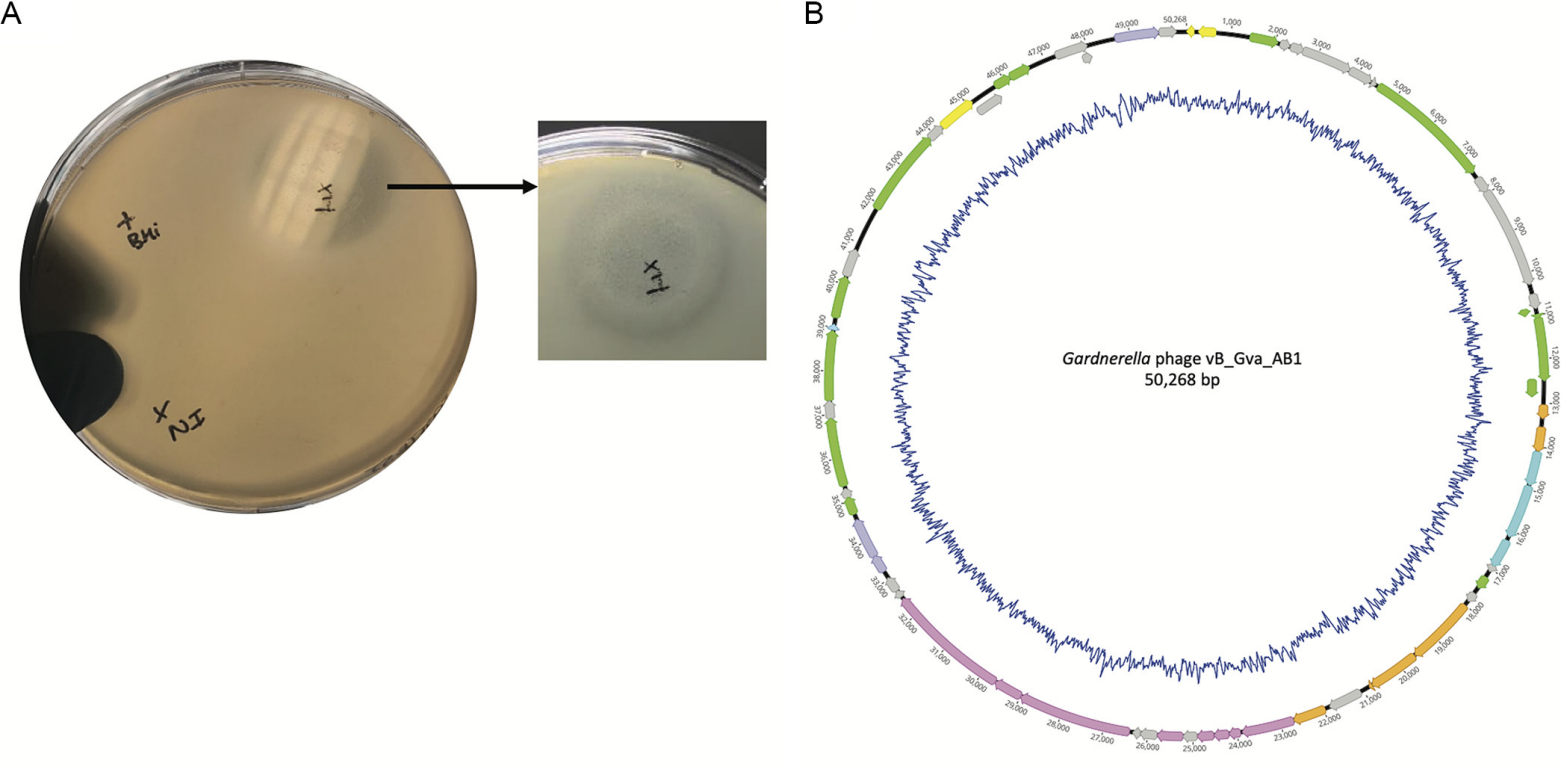
Double-layer plaque assay and circular genome representation of the *Gardnerella* phage vB_Gva_AB1. (A) Spot test showing large double turbid halo phage plaque, characteristic of temperate phages from induced culture (I), not-induced culture (NI), and control of culture (supplemented BHI [sBHI] medium only). (B) Genome organization with predicted ORF direction and annotation colored according to functional group of genes: yellow, lysogeny; orange, DNA packaging; green, DNA replication, transcription, and repair; pink, viral particle structure; light blue, phage virulence against host; purple, lysis; and gray, hypothetical protein. GC content is represented by the central dark-blue line. Genome visualization was obtained using Geneious software v2021.0.1.

Gardnerella vaginalis strain CSUR P680, isolated from a vaginal swab of a French woman suffering from BV (IHU Méditerranée Infection, Marseille, France), was used to induce prophages. The patient provided informed consent, and the study was authorized by the ethics committee of the Institut Federatif de Recherche 48 (number 09-022). The vaginal swab was treated as previously described (12), and the strain was cultured in brain-heart infusion (BHI; BD) broth medium, supplemented with yeast extract (0.5%; Fisher Scientific), glucose (0.25%; Euromedex), and fetal bovine serum (10%; Life Technologies) at 37°C, 5% CO_2_ (BD), and agitation (200 rpm). Bacteriophage excision was induced by adding mitomycin C (10 μg/ml; MedChemExpress). The overnight culture was centrifuged (13,000 × *g*; 10 min), and the supernatant was filtered (0.22 μm; Dutscher). On a Gardnerella vaginalis P680 double-layer plaque assay (0.6% agar; Life Technologies), a large double turbid halo phage plaque, characteristic of temperate phages (Fig. 1A), was observed. Phage DNA extraction was performed on 1 ml of clarified and filtered induced culture supernatant by using a High Pure viral nucleic acid large-volume kit (Roche), randomly amplified using the GenomiPhi v3 DNA amplification kit (GE Healthcare), purified with a NucleoFast plate (Macherey-Nagel), and quantified using a Qubit double-stranded DNA (dsDNA) high-sensitivity assay kit (Life Technologies) and instrument (Invitrogen), all according to the manufacturer’s protocols. Purified nucleic acids were used to construct a paired-end library with the Nextera XT library kit and sequenced with MiSeq technology (Illumina, Inc.). Raw paired reads (2,594,168) were imported, trimmed, and *de novo* assembled using the CLC Genomics Workbench v7.5 software. All tools were run with default parameters unless otherwise specified. This generated 1,916 contigs with a length ranging from 53 bp to 23,344 bp and a coverage of 1× to 11,936×. Contigs with a length of >1,000 bp and a coverage of >1,000× were manually concatenated according to their position on a bacterial reference genome (GenBank accession number CP019058.1) using BLASTn. Concatenation and circularization of the genome were verified, corrected, and validated by PCR performed with AmpliTaq Gold 360 master mix (Life Technologies) and Sanger sequencing using the BigDye Terminator v1.1 cycle sequencing kit (Life Technologies) and a 3500xL genetic analyzer capillary sequencer (Thermo Fisher) according to the manufacturers’ recommendations.

The complete genome consisted of a circular double-stranded DNA molecule of 50,268 bp with a GC content of 39.55% (Fig. 1B). The closest viral homolog (76.42% identity and 3% query coverage) detected after a BLASTn search against the nonredundant (nr) database was *Streptococcus* phage Javan112 (GenBank accession number MK448843.1), which belongs to the *Siphoviridae* family. A total of 62 open reading frames (ORFs) were predicted with GeneMarkS v4.28 (13, 14). No tRNAs were detected using tRNAscan-SE 2.0 (15, 16). No CRISPR spacer arrays, identified within *G. vaginalis* complete genomes available in the NCBI genome database using CRISPRCasFinder (17), perfectly matched (100% query coverage and 100% identity) the *Gardnerella* phage vB_Gva_AB1 genome sequence. The genome was manually annotated using BLASTp against the nr database and InterPro. Putative function was assigned to the majority (64.5%) of predicted ORFs, whereas 35.5% matched hypothetical proteins or protein domains that had no characterized function. InterPro provided functional annotation for ORF33 (HK97 gp10 family phage protein) and ORF38 (phage tail tape measure protein). Predicted ORFs were categorized in 7 modules—lysogeny, DNA packaging, DNA replication/transcription/repair, viral particle structure, phage virulence against host, host lysis, and hypothetical protein (Fig. 1B, Table 1).

**TABLE 1:** Characteristics of *Gardnerella* phage vB_gva_AB1 ORFs and predicted functions^*a*^

ORF	Strand	ORF start (bp)	ORF end (bp)	aa length	Description (best BLASTn hit)	Maximum score	Total score	Query coverage (%)	E value	Identity (%)	GenBank accession no.	Functional group
1	−	1	162	53	IS*3* family transposase	107	107	100	6.00e-29	92.45	WP_116431825.1	Lysogeny
2	−	256	666	136	IS*3* family transposase	276	276	100	7.00e-93	97.79	WP_103014070.1	Lysogeny
3	+	1433	2065	210	RNA polymerase subunit sigma-24	421	421	100	9.00e-149	97.14	RFT26841.1	DNA replication/transcription/repair
4	+	2126	2347	73	Hypothetical protein	151	151	100	3.00e-46	100.00	WP_016815920.1	Hypothetical protein
5	+	2360	2662	100	Hypothetical protein	195	195	100	8.00e-63	99.00	WP_014554450.1	Hypothetical protein
6	+	2655	3785	376	DUF2800 domain-containing protein	752	752	99	0	96.53	WP_014554451.1	Hypothetical protein
7	+	3787	4335	182	DUF2815 family protein	370	370	100	2.00e-129	98.35	WP_018645384.1	Hypothetical protein
8	+	4349	4459	36	Hypothetical protein	70.5	70.5	100	4.00e-15	97.22	OKY54901.1	Hypothetical protein
9	+	4521	7424	967	DNA polymerase	1,983	1,983	100	0	98.55	WP_048730082.1	DNA replication/transcription/repair
10	+	7578	7952	124	Hypothetical protein	255	255	100	1.00e-85	95.97	RFT36085.1	Hypothetical protein
11	+	7945	10278	777	Hypothetical protein	1,594	1,594	100	0	98.07	WP_116440744.1	Hypothetical protein
12	+	10507	10842	111	Hypothetical protein	224	224	100	1.00e-73	98.20	WP_004117127.1	Hypothetical protein
13	+	10814	10975	53	VRR-NUC domain-containing protein	90.9	90.9	84	3.00e-22	100.00	WP_076002715.1	DNA replication/transcription/repair
14	+	10981	11073	30	VRR-NUC domain-containing protein	61.2	61.2	100	6.00e-11	100.00	WP_102155316.1	DNA replication/transcription/repair
15	+	11054	12493	479	DEAD/DEAH box helicase	984	984	100	0	99.16	WP_103014033.1	DNA replication/transcription/repair
16	+	12453	12881	142	RNA polymerase subunit sigma-70	287	287	100	5.00e-98	100.00	WP_101887643.1	DNA replication/transcription/repair
17	+	13034	13366	110	HNH endonuclease	227	227	100	4.00e-75	98.18	WP_020760705.1	DNA packaging
18	+	13555	14106	183	Terminase	377	377	100	4.00e-132	99.45	WP_032842293.1	DNA packaging
19	+	14119	14904	261	*S*-Adenosylmethionine synthetase	533	533	100	0	100.00	WP_014554462.1	Phage virulence against host
20	+	14919	16154	411	DNA modification methylase	852	852	100	0	99.51	RIY23021.1	Phage virulence against host
21	+	16253	16903	216	Virulence protein	440	440	100	9.00e-156	100.00	WP_112928563.1	Phage virulence against host
22	+	16887	17051	54	Hypothetical protein	100	100	100	2.00e-26	92.59	WP_165847508.1	Hypothetical protein
23	+	17217	17501	94	Transcriptional regulator	189	189	100	2.00e-60	98.94	WP_076002844.1	DNA replication/transcription/repair
24	+	17651	17875	74	Hypothetical protein	154	154	100	3.00e-47	100.00	WP_112928565.1	Hypothetical protein
25	+	18000	19592	530	Terminase large subunit	1,101	1,101	100	0	98.87	WP_116691915.1	DNA packaging
26	+	19602	20774	390	Phage portal protein	782	782	97	0	97.64	WP_076002846.1	DNA packaging
27	+	20765	20848	27	Portal protein	56.2	56.2	100	2.00e-09	100.00	RIY25730.1	DNA packaging
28	+	21078	21845	255	Hypothetical protein	513	513	100	0	98.04	WP_004104990.1	Hypothetical protein
29	+	21962	22705	247	ATP-dependent Clp protease proteolytic subunit	511	511	100	0	100.00	WP_192382072.1	DNA packaging
30	+	22708	23892	394	Phage major capsid protein	803	803	100	0	99.49	RIY23012.1	Viral particle structure
31	+	23918	24196	92	Phage gp6-like head-tail connector protein	188	188	100	4.00e-60	100.00	WP_004125785.1	Viral particle structure
32	+	24197	24529	110	Phage head closure protein	224	224	100	6.00e-74	98.18	WP_076002725.1	Viral particle structure
33	+	24526	24906	126	Hypothetical protein	241	241	100	2.00e-80	98.41	RFT26873.1	Viral particle structure
34	+	24899	25228	109	Hypothetical protein	222	222	100	4.00e-73	99.08	WP_103014101.1	Hypothetical protein
35	+	25221	25793	190	Tail protein	386	386	100	2.00e-135	100.00	WP_014554473.1	Viral particle structure
36	+	25811	26176	121	Hypothetical protein	244	244	100	2.00e-81	99.17	WP_014554474.1	Viral particle structure
37	+	26173	26337	54	Hypothetical protein	111	111	100	9.00e-31	100.00	AEF31840.1	Hypothetical protein
38	+	26419	28986	855	Hypothetical protein	1,704	1,704	100	0	99.77	WP_004125799.1	Viral particle structure
39	+	28989	29651	220	Phage tail component protein	453	453	100	7.00e-161	98.64	EPI45861.1	Viral particle structure
40	+	29652	32471	939	Phage tail protein	1,927	1,927	99	0	99.04	WP_076002853.1	Viral particle structure
41	+	32517	32681	54	Hypothetical protein	106	106	100	1.00e-28	100.00	EPI54250.1	Hypothetical protein
42	+	32684	33043	119	Hypothetical protein	223	223	100	3.00e-73	96.64	EIK79881.1	Hypothetical protein
43	+	33206	33625	139	Phage holin family protein	283	283	100	3.00e-96	100.00	WP_032812430.1	Lysis
44	+	33618	34538	306	1,4-beta-*N*-Acetylmuramidase	606	606	100	0	95.75	WP_065189413.1	Lysis
45	+	34688	35095	135	RNA polymerase subunit sigma-70	276	276	100	9.00e-94	99.26	WP_103014084.1	DNA replication/transcription/repair
46	+	35082	35294	70	Hypothetical protein	142	142	100	1.00e-42	98.57	WP_111822612.1	Hypothetical protein
47	+	35356	36924	522	Recombinase family protein	1,050	1,050	100	0	97.32	WP_019260895.1	DNA replication/transcription/repair
48	+	36921	37331	136	Hypothetical protein	274	274	100	6.00e-93	99.26	WP_020760681.1	Hypothetical protein
49	+	37333	38910	525	Recombinase family protein	1,076	1,076	100	0	99.05	WP_004125822.1	DNA replication/transcription/repair
50	+	38932	39072	46	Hypothetical protein	NA	NA	NA	NA	NA	NA	Hypothetical protein
51	+	39227	40171	314	Recombinase family protein	638	638	100	0	99.36	WP_119675718.1	DNA replication/transcription/repair
52	+	40191	40829	212	Hypothetical protein	434	434	100	8.00e-154	100.00	WP_119675717.1	Hypothetical protein
53	+	41857	43866	669	ATP-binding cassette domain-containing protein	1,352	1,352	100	0	100.00	WP_014554440.1	DNA replication/transcription/repair
54	+	43863	44255	130	Hypothetical protein	258	258	100	1.00e-86	100.00	WP_004114689.1	Hypothetical protein
55	+	44242	45132	296	Lantibiotic transport ATP-binding protein	582	582	100	0	98.65	EIK77256.1	Lysogeny
56	+	45101	45751	216	Hypothetical protein	420	420	100	5.00e-148	100.00	AEF31611.1	Hypothetical protein
57	+	45755	46144	129	Thioredoxin family protein	267	267	100	2.00e-90	100.00	WP_004125685.1	DNA replication/transcription/repair
58	+	46150	46623	157	Thioredoxin family protein	319	319	100	5.00e-110	99.36	WP_004125686.1	DNA replication/transcription/repair
59	+	47265	47828	187	Hypothetical protein	380	380	100	2.00e-133	100.00	WP_004114700.1	Hypothetical protein
60	+	47842	48066	74	Hypothetical protein	149	149	100	6.00e-45	100.00	EPI41518.1	Hypothetical protein
61	+	48651	49661	336	Amidase domain-containing protein	703	703	100	0	100.00	WP_048730094.1	Lysis
62	+	49648	50052	134	Hypothetical protein	261	261	100	8.00e-88	100.00	WP_048730092.1	Hypothetical protein

a NA, not applicable; +, positive; −, negative; bp, base pair; aa, amino acid.

### Data availability.

The data set was deposited in the Sequence Read Archive (SRA) under the number SRX9776169 (BioProject number PRJNA687336). The complete and annotated genome sequence is available under GenBank accession number MW387018.1.

## References

[1] Gardner HL, Dukes CD. 1955. Haemophilus vaginalis vaginitis: a newly defined specific infection previously classified non-specific vaginitis. Am J Obstet Gynecol 69:962–976. doi:10.1016/0002-9378(55)90095-8. 14361525

[2] Morrill S, Gilbert NM, Lewis AL. 2020. Gardnerella vaginalis as a cause of bacterial vaginosis: appraisal of the evidence from in vivo models. Front Cell Infect Microbiol 10:168. doi:10.3389/fcimb.2020.00168. 32391287 PMC7193744

[3] Workowski KA, Berman S, Centers for Disease Control and Prevention. 2010. Sexually transmitted diseases treatment guidelines, 2010. MMWR Recomm Rep 59:1–110. https://pubmed.ncbi.nlm.nih.gov/21160459/. 16888612

[4] Bradshaw CS, Morton AN, Hocking J, Garland SM, Morris MB, Moss LM, Horvath LB, Kuzevska I, Fairley CK. 2006. High recurrence rates of bacterial vaginosis over the course of 12 months after oral metronidazole therapy and factors associated with recurrence. J Infect Dis 193:1478–1486. doi:10.1086/503780. 16652274

[5] Mastromarino P, Hemalatha R, Barbonetti A, Cinque B, Cifone MG, Tammaro F, Francavilla F. 2014. Biological control of vaginosis to improve reproductive health. Indian J Med Res 140:91. PMC4345761 25673551

[6] Hill C, Guarner F, Reid G, Gibson GR, Merenstein DJ, Pot B, Morelli L, Canani RB, Flint HJ, Salminen S, Calder PC, Sanders ME. 2014. Expert consensus document. The International Scientific Association for Probiotics and Prebiotics consensus statement on the scope and appropriate use of the term probiotic. Nat Rev Gastroenterol Hepatol 11:506–514. doi:10.1038/nrgastro.2014.66. 24912386

[7] Martinez RCR, Franceschini SA, Patta MC, Quintana SM, Gomes BC, De Martinis ECP, Reid G. 2009. Improved cure of bacterial vaginosis with single dose of tinidazole (2 g), Lactobacillus rhamnosus GR-1, and Lactobacillus reuteri RC-14: a randomized, double-blind, placebo-controlled trial. Can J Microbiol 55:133–138. doi:10.1139/w08-102. 19295645

[8] Bradshaw CS, Pirotta M, De Guingand D, Hocking JS, Morton AN, Garland SM, Fehler G, Morrow A, Walker S, Vodstrcil LA, Fairley CK. 2012. Efficacy of oral metronidazole with vaginal clindamycin or vaginal probiotic for bacterial vaginosis: randomised placebo-controlled double-blind trial. PLoS One 7:e34540. doi:10.1371/journal.pone.0034540. 22509319 PMC3317998

[9] Heczko PB, Tomusiak A, Adamski P, Jakimiuk AJ, Stefański G, Mikołajczyk-Cichońska A, Suda-Szczurek M, Strus M. 2015. Supplementation of standard antibiotic therapy with oral probiotics for bacterial vaginosis and aerobic vaginitis: a randomised, double-blind, placebo-controlled trial. BMC Womens Health 15:115. doi:10.1186/s12905-015-0246-6. 26635090 PMC4669640

[10] Ravat F, Jault P, Gabard J. 2015. Bactériophages et phagothérapie: utilisation de virus naturels pour traiter les infections bactériennes. Ann Burns Fire Disasters 28:13–20. 26668557 PMC4665175

[11] Malki K, Shapiro JW, Price TK, Hilt EE, Thomas-White K, Sircar T, Rosenfeld AB, Kuffel G, Zilliox MJ, Wolfe AJ, Putonti C. 2016. Genomes of Gardnerella strains reveal an abundance of prophages within the bladder microbiome. PLoS One 11:e0166757. doi:10.1371/journal.pone.0166757. 27861551 PMC5115800

[12] Lagier J-C, Hugon P, Khelaifia S, Fournier P-E, La Scola B, Raoult D. 2015. The rebirth of culture in microbiology through the example of culturomics to study human gut microbiota. Clin Microbiol Rev 28:237–264. doi:10.1128/CMR.00014-14. 25567229 PMC4284300

[13] Besemer J, Lomsadze A, Borodovsky M. 2001. GeneMarkS: a self-training method for prediction of gene starts in microbial genomes. Implications for finding sequence motifs in regulatory regions. Nucleic Acids Res 29:2607–2618. doi:10.1093/nar/29.12.2607. 11410670 PMC55746

[14] Borodovsky M, Lomsadze A. 2011. Gene identification in prokaryotic genomes, phages, metagenomes, and EST sequences with GeneMarkS suite. Curr Protoc Bioinformatics Chapter 4:Unit 4.5.1–4.5.17. doi:10.1002/0471250953.bi0405s35. 21901741

[15] Chan PP, Lowe TM. 2019. tRNAscan-SE: searching for tRNA genes in genomic sequences. Methods Mol Biol 1962:1–14. doi:10.1007/978-1-4939-9173-0_1. 31020551 PMC6768409

[16] Lowe TM, Chan PP. 2016. tRNAscan-SE On-line: integrating search and context for analysis of transfer RNA genes. Nucleic Acids Res 44:W54–W57. doi:10.1093/nar/gkw413. 27174935 PMC4987944

[17] Couvin D, Bernheim A, Toffano-Nioche C, Touchon M, Michalik J, Néron B, Rocha EPC, Vergnaud G, Gautheret D, Pourcel C. 2018. CRISPRCasFinder, an update of CRISRFinder, includes a portable version, enhanced performance and integrates search for Cas proteins. Nucleic Acids Res 46:W246–W251. doi:10.1093/nar/gky425. 29790974 PMC6030898

